# A Comprehensive Investigation of the Current Subacute Sclerosing Panencephalitis (SSPE) Treatment Options to Improve Patient Quality of Life

**DOI:** 10.7759/cureus.28389

**Published:** 2022-08-25

**Authors:** Ariana Pritha, Tanisha N Medha, Ravindra K Garg

**Affiliations:** 1 Neurosciences, The University of New Mexico, Albuquerque, USA; 2 Medicine, University of New Mexico, Albuquerque, USA; 3 Neurology, King George's Medical University, Lucknow, IND

**Keywords:** inosine pranobex, prolonging life, ribavirin, interferon, sspe current treatments, subacute sclerosing panencephalitis

## Abstract

Subacute sclerosing panencephalitis (SSPE) is a progressive, disabling, and deadly neurological disorder related to measles (rubeola) infection occurring primarily in children. The slow but persistent viral infection occurs in children or young adults and affects their central nervous system (CNS). There have been plenty of reports on SSPE throughout the world, but it is considered a rare disease in developed countries. This research focuses on comparing the current treatments available to prolong the life of patients for over three years after the onset of SSPE. The goal was to identify possible patterns or trends among the treatments in order to find the best possible method to lengthen a patient's life. The results indicated that interferon alpha, inosine pranobex, and ribavirin display the most effective treatment plan and indicate the most potential in discovering a more effective therapeutic for SSPE.

## Introduction and background

Even after the creation of the measles-mumps-rubella (MMR) vaccine, this prophylactic treatment still has not managed to eradicate the rare pediatric neurodegenerative disease known as subacute sclerosing panencephalitis (SSPE) [1]. Following years of research, a cure has yet to be presented, and treatments are controversial as large-scale trials cannot confirm reports due to the rarity of the disease. As more measles outbreaks occur, there are more SSPE cases arising in developed regions like the United Kingdom due to the misconception of the measles vaccine being linked to autism [2-3]. In the near future, there will be more demand for treatment plans for SSPE, which is the driving force for this report which looks at recent publications and the more successful options. Although none of the treatments will provide a cure, the discussion will be centered around treatments that increase the quality of living and prolong the lives of patients.

## Review

Objectives

The primary objective of this study is to record the recent epidemiology of SSPE treatment from 1999 to 2022. The secondary objective is to find patterns and trends from all treatment cases.

Method

Articles were researched through the PubMed, Embase, and Google Scholar databases using the keywords of “SSPE treatments”, and later “SSPE” if the search results were lacking. The papers were further filtered, with the earliest reports dating back from 1999 and the most recent up to 2022. The resulting papers’ abstracts were analyzed to assess the relevance of the papers to the topic of this review. The treatment would be considered successful if the patient survives for longer than three years. 

Eligibility Criteria

The papers must be accessible, in English, and the data must be in standard units. Papers that were excluded tended to be irrelevant to the main topic.

Search Strategy

**Figure 1:**
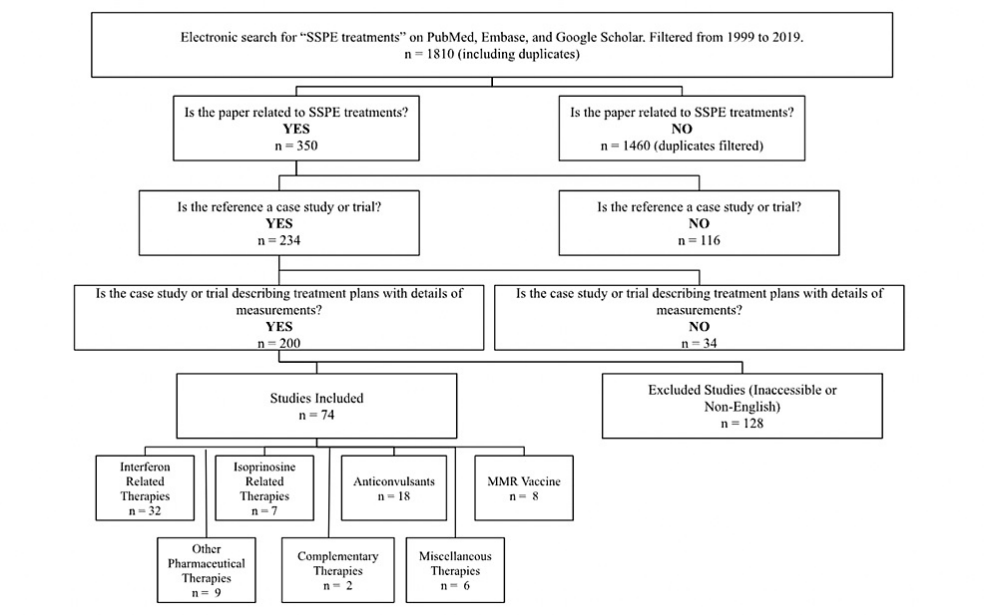
Flowchart of search strategy to visualize the process of filtration of articles The total number of studies is 74, but the total n for treatment options is 80, since some studies reported on multiple treatment plans/ mixed treatment plans [1-25] SSPE - subacute sclerosing panencephalitis, MMR - measles-mumps-rubella

The search strategy (outlined in Figure 1) was used to filter the studies from the most recent and relevant to the topic of SSPE treatments. The only obstacles were the tagged dates for the articles would be more recent, whereas many of the articles were published before 1999, and from the plethora of results found, the majority were not relevant to the topic of SSPE. Unfortunately, many articles were inaccessible due to the inability to translate from different languages. There was not much progress in novel studies from 2019 to 2022 due to the SARS-CoV-2 pandemic.

Search Results

Search results were recognized as the papers discussed in this review, but in terms of analyzing data, only the case studies and randomized clinical trials (RCTs) were implemented in the results section to make direct references. 

Study Characteristics

The studies chosen were case studies, randomized controlled trials, and reviews to observe the progression of the treatment, as therapy varies from person to person, and many treatments are thought to be controversial. The studies were picked and sorted following the search strategy and categorized based on the number of articles found for each type of treatment. The studies were grouped by therapies for prolonging life and symptomatic treatments. Treatments that were considered miscellaneous were not reported much in the past twenty years or did not prove to be very successful despite having potential for the future. 

Study Quality 

**Figure 2:**
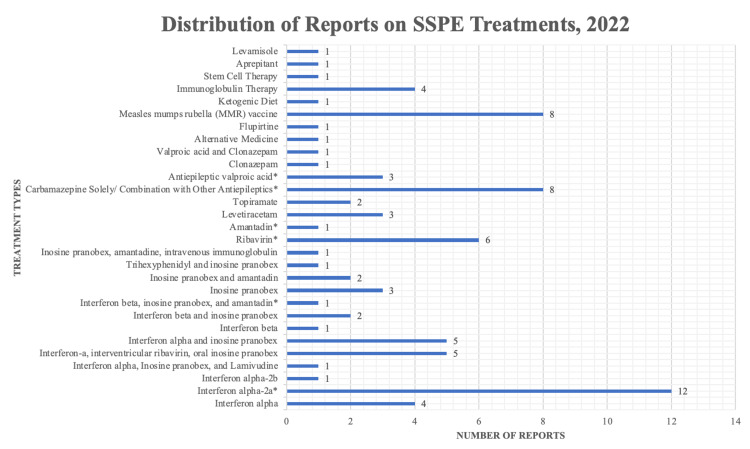
Graph of distribution of studies, highlighting the more common treatment types that are being reported SSPE - subacute sclerosing panencephalitis

Due to some references being literature reviews, not all the outlined studies will be displayed in the results section (Table 1, Figure 2). The papers that were chosen all have a general consensus of prophylactic treatment being the “cure” for this devastating disease; however, the bias is included when there is a qualitative assessment of the patient, such as assessing behavior. There was definitely a strong bias in many of the interferon-related therapy papers before 1999 due to the results being controversial [3]. The papers now are very suggestive, and the bias is often worded in a way that invokes further study using improved methods. Overall, the study quality is very reliable as many papers recent to the date the papers are published can support the claim with similar findings despite having different conclusions. There is always a risk of bias, but in the case of the studies chosen for this review, the risk is very low.

**Table 1:** Number of articles referenced for each topic RCTS - randomized controlled studies, MMR - measles-mumps-rubella * indicates that it is a duplicated report as a previous report was referenced for data ** not included in the results section (may be used for reference in discussion)

Intervention groupings	No. of studies/RCTS/ reviews referenced
Interferon-related therapies	
Interferon alpha	4
Interferon alpha-2a*	12
Interferon alpha-2b	1
Interferon alpha, inosine pranobex, and lamivudine	1
Interferon-a, interventricular ribavirin, oral inosine pranobex	5
Interferon alpha and inosine pranobex	5
Interferon beta	1
Interferon beta and inosine pranobex	2
Interferon beta, inosine pranobex, and amantadin*	1
Inosine pranobex related therapies	
Inosine pranobex	3
Inosine pranobex and amantadin	2
Trihexyphenidyl and inosine pranobex	1
Inosine pranobex, amantadine, intravenous immunoglobulin	1
Other pharmaceutical therapies	
Ribavirin*	6
Amantadin*	1
Aprepitant	1
Levamisole	1
Anticonvulsants	
Levetiracetam	3
Topiramate	2
Carbamazepine solely/ combination with other antiepileptics*	8
Antiepileptic valproic acid*	3
Clonazepam	1
Valproic acid and clonazepam	1
Complementary therapies	
Alternative medicine	1
Flupirtine	1
Measles (MMR) vaccination**	
Measles mumps rubella (MMR) vaccine	8
Miscellaneous treatments	
Ketogenic diet	1
Immunoglobulin therapy	4
Stem cell therapy	1
Total case studies: 74	

Results

Upon the analysis of 74 reports, the collected data has been filtered, sorted by most relevant, and organized into groups to aid in the observations of trends in therapies that potentially are more successful than others. Data was collected from selected papers and included case studies and trial data. The data is organized by category and will be extrapolated to find some ubiquitous global trends in SSPE patients, as the origin of the paper was disregarded.

Interferon Therapies

Interferon is a natural substance released by the immune system when there is a viral infection present. The pharmaceutical drug is that same compound and induces cells’ antiviral defense via releasing proteins to attack the disease. Interferon is most commonly used against leukemia, but this form of immunotherapy has become one of the most common treatment options despite there being no protocol for SSPE treatments (Table 2).

**Table 2:** Randomized data collection for interferon-related therapies IFNb - interferon beta, IFN - interferon

Type of interferon treatment	Reference	Sex/ age of onset	Patient SSPE stage before treatment according to Jabbour classification (numbers indicate the number of patients)	Dosage of treatment	Progress of treatment
Interferon alpha	Nasirian et al., 2016 [4]	15 patients: 11 males (71%) and 4 females (29%), mean age: 9	Stage I: 4	Subcutaneous injection of interferon alpha: 3-6 million units, three times weekly for three months	Two cases reached cessation. Five cases had slowed progression. Among those whose condition slowed progression, two died between two to three years after admission, and three lived for between three to five years. In eight patients the treatment was ineffective.
			Stage II: 6		
			Stage III: 5		
	Miyazaki et al., 2005 [5]	Male/8	Stage IIA	Initial dose: 1 × 10^6^ IU weekly for the next six months. Dose change after six months: 1.5 × 10^6^ IU weekly. Dose change after six months: 3 × 10^6^ IU weekly. Last increase: 5 × 10^6^ IU IFNa weekly and 6 × 10^6^ IU of IFNb weekly	Initial dose showed no remarkable effects. Dose change after six months: the patient could run and speak short phrases. He could do very simple math, complete up to secondary education, and could complete tasks like dressing himself. Dose change after six months: condition deteriorated to stage IIB. Last increase: no remarkable benefit and interferon therapy was ceased. Deteriorated condition to stage IV
Interferon alpha-2a	Moodley et al., 2015 [6]	Female/19	Stage II	Intraventricular interferon alpha: 1.5 million units on alternate days for six weeks	Showed progression as she could properly communicate. The treatment was discontinued when she tested positive for HIV as the effects of the drug were unknown.
Interferon alpha-2b	Campbell et al., 2005 [7]	Female/6	Stage III	Two weeks: 1 million units daily; six weeks: weekly; three months: every other week	Condition deteriorated further
Interferon alpha, inosine pranobex, and lamivudine	Aydin et al., 2003 [8]	N/A	N/A: 19	Oral inosine pranobex: 100 mg/kg/day for six months. interferon alpha-2a: 10 m U/m2/three times a week for six months. Oral lamivudine: 10 mg/kg daily for six months	Mortality rate: 3 (15.7%), remission rate: 7 of 19 (36.8%), mean survival period longer than the control group.
Control for Interferon alpha, inosine pranobex, and lamivudine		N/A	N/A: 13	No dose	Mortality rate: 6 (46%), remission Rate: 0 of 13 (0%), mean survival period shorter than the treatment group.
Interferon alpha, ribavirin, inosine pranobex	Hosoya et al., 2011 [9]	Male/15	Between stage III and stage IV	Ribavirin 1 mg/kg twice a day. Oral inosine pranobex daily	Stage III, improved condition
		Female/14	Stage II	Ribavirin 1 mg/kg twice a day repeated for more than six months. Oral inosine pranobex daily	Stage I, improved condition
		Male/6	Stage III	IFN-therapy at a dose of 300 × 10^4 ^IU twice a week for 20 months. Intraventricular ribavirin administration at a dose of 1 mg/kg three times a day for 11 months. Oral inosine pranobex daily	Stage III, stable
		Female/6	Between stage III and stage IV	IFN-therapy at a dose of 300 × 10^4^ IU twice a week for 13 months. Intraventricular ribavirin administration at doses of 1, 2, and 3 mg/kg once a day for 12 months. Oral inosine pranobex daily	Stage III, improved condition
		Male/11	Stage III	IFN-therapy at a dose of 300 × 10^4^ IU twice a week. Intraventricular ribavirin administration at a dose of 1 mg/kg twice a day. Oral inosine pranobex daily	Stage III, stable
	Ohya et al., 2014 [10]	Female/15	Stage I	Combination therapy by intraventricular interferon alpha and ribavirin	Lived for over three years and attended school with the help of a special needs teacher.
		Female/12	Stage II	Inosine pranobex: 100 mg/kg daily. Combination therapy by intraventricular interferon alpha and ribavirin. Treatment for more than three years	Stage II, but improved as she can communicate and attends school with a special needs teacher.
Interferon alpha and inosine pranobex	Gokcil et al., 1999 [11]	Male/21	Stage IIA	Six weeks (repeated in two to six month intervals): intraventricular interferon-alpha administered at 1 × 10^5^ U/m^2^ and increased to 1 × 10^6^ U/m^2^ of body area daily for five days a week. Daily: inosine pranobex dose of 50 to 100 m/kg of body mass daily	Stage IIB, deteriorated
		Male/20	Stage IIB		Stage IIB, stable
		Male/18	Stage IIA		Stage I, improved condition
		Male/22	Stage IIA		Stage IIA, stable
	Kwak et al., 2019 [12]	Male/13	Stage III	Oral inosine pranobex: 100 mg/kg/day in three divided doses and intraventricular interferon alpha: 1×106 u^2^ twice a week and escalating to five days a week after six months	Stage III for thirteen years. Survived in a bedridden state and can communicate with indiscernible “babble”.
	Solomon et al., 2002 [13]	Male/17	Stage II	Intraventricular interferon alpha: beginning at 100,000 U/m^2^/day, increasing to 1 million U/m^2^/day), ribavirin (60 mg/kg/day intravenously), inosine pranobex (3 g/day)	Showed progress as he returned home and began interacting more socially, but 10 months later he fell into a vegetative state (Stage IV) and died
Interferon beta and inosine pranobex	Takashima et al., 2003 [14]	Female/20	Stage II	Inosine pranobex: 70 mg per kg of body mass a month Intraventricular Interferon beta: 1.0 million international units per square meter (MIU/m^2^)	Stage II, very little improvement according to the study, but the general situation remained the same.
	Har-Even et al., 2011 [15]	Male/16	Stage I	Oral inosine pranobex: 1 g three times per day. Subcutaneous Interferon-b-1a: 22 μg three times per week and later increased to 44 μg three times per week	Rapid progression to stage II then stage IV and then died after one year of hospitalization.

Inosine Pranobex-Related Therapies

Inosine pranobex, also known by the trade name Isoprinosine, is an immunostimulant. In other words, it has the same function as interferons. It is commonly used to subdue SSPE symptoms as it is a synthetic compound that inhibits measles (and a majority of viral) RNA synthesis and replication (Table 3).

**Table 3:** Data collection for inosine pranobex-related therapies SSPE - subacute sclerosing panencephalitis

Type of inosine pranobex-related treatment	Reference	Sex and age of onset (in years unless otherwise stated)	Patient SSPE stage	Dosage of treatment	Progress of treatment
Inosine pranobex	Campbell et al., 2005 [7]	Female/6	Stage I	100 mg/kg divided qid	Treatment discontinued and the patient slowly deteriorated to stage II. Died from a respiratory illness
	Gokcil et al., 1999 [11]	Male/22	Stage IIB	50 to 100 mg/kg of body mass daily	Stage IIA, improved condition
		Male /21	Stage IIA		Stage IIC, deteriorated condition
		Male/18	Stage IIB		Stage IIC, deteriorated condition
		Male/21	Stage IIB		Stage IIC, deteriorated condition
	Cruzado et al., 2002 [16]	Female/18 months	Stage II	100 mg/kg per day	Vegetative at 20 months and died at 28 months
	Bobele et al., 1999 [17]	Male/5	Stage III	100 mg/kg/day orally for six months	Improved to saying three single words, responding to visual and auditory stimuli, and attempting to sit.
	Nasirian et al., 2016 [4]	15 patients: 11 males (71%) and 4 females (29%), mean age: 9	Stage I: 4	100 mg/kg/day of Inosine pranobex (Inosine pranobex) for six months	In four cases the disease progression stopped. Six cases exhibited slow progression and in six others the drug had no effect. Of the six patients who exhibited slow progression, after admission of the drug three lived for four extra years and two for up to seven years while one lives ten years after treatments
			Stage II: 5		
			Stage III: 5		
			Stage III: 2		

Other Pharmaceutical Therapies

The following drugs are often combined, but the individual compounds may provide insight into how different drugs react in the process of SSPE (Table 4).

**Table 4:** Data collection for other pharmaceutical therapies SSPE - subacute sclerosing panencephalitis, CSF - cerebrospinal fluid, HI - hemagglutination inhibition

Other pharmaceutical therapies	Reference	Sex/ age at onset	Patient SSPE stage	Dosage of treatment	Progress of treatment
Ribavirin	Campbell et al., 2005 [7]	Female/6	Stage I	1 mg/kg twice a day	Treatment discontinued and the patient slowly deteriorated to stage II. Died from a respiratory illness
	Tomoda et al., 2003 [18]	10 patients: 5 males and 5 females, mean age: 12.1	Stage I: 2	Intraventricular administration: 1 to 7.7 mg/kg	Seven patients showed decreased CSF measles HI antibodies, two showed no change and one showed an increase. Slow progression was observed for patients during the study period; six patients improved clinically and one improved to the first stage of Jabbour’s classification
			Stage II: 6		
			Stage III: 1		
			Stage IV: 1		
	Bobele et al., 1999 [17]	Male/5	Stage III	Intravenous ribavirin: 20 mg/kg daily for three weeks	He was discharged in a vegetative state (stage IV)
Amantadin	Nasirian et al., 2016 [4]	15 patients: 11 males (71%) and 4 females (29%), mean age: 9	Stage I: 3	Oral Amantadin administration: 10-15 mg/kg for three to six months	1 patient: full cessation, 3 patients: slowed progression; two lived between two to three years after onset, and the third lived over three years, 10 patients: non-effective treatment
			Stage II: 4		
			Stage III: 6		
	Bobele et al., 1999 [17]	Male/5	Stage III	Treated for 21 days; dosage not specified	MRI findings found no improvement
Aprepitant	Oncel et al., 2020 [19]	Patients: 62, group 1 median age (tested with aprepitant): 18, placebo group median age: 22, sex: unknown (double-blind and randomized)	N/A	Oral aprepitant administration: 250 mg/day for 15 days with an interval of two months between courses	27 patients: left clinical trial (within a year). Both groups: an increase in cerebral atrophy on MRI was observed. Placebo group: measles-specific immunoglobulin G index decreased
Levamisole	Panda et al., 2020 [20]	Contracted measles between six to eight years, female/21	Stage III	Oral levamisole administration: N/A with a gradual increase in dosage	After two months, the patient experienced a slight decrease in major jerks. Between three and six months, both periodic myoclonus and major jerks had noticeably decreased. After 20 months, the patient had myoclonus. By 21 months, the patient had subsided myoclonus

Anticonvulsants 

These treatments are used to subdue the myoclonic jerks and seizures that patients diagnosed with SSPE may have (Table 5). The most common anticonvulsant is carbamazepine for seizures, but it is used as a last resort as it does not halt myoclonic seizures. Trihexyphenidyl is primarily used to battle the side effects of antipsychotic drugs, but as the body adjusts to the dose, more is needed for it to continue to be effective [8]. Valproic acid is primarily used to stop absence seizures, which is also why it is paired with other anticonvulsants [9]. Clonazepam is a tranquilizer that is also used to treat seizures and relieves pain [10].

**Table 5:** Data collection for anticonvulsants for myoclonic jerks and seizures SSPE - subacute sclerosing panencephalitis

Anticonvulsants	Reference	Sex/ age at onset	Patient SSPE stage	Dosage of treatment	Progress of treatment
Carbamazepine	Solomon et al, 2002 [13]	Male/17	Stage II	N/A	Successfully subdued seizures while on this treatment.
	Har-Even et al., 2011 [15]	Male/16	Stage I	N/A	Was used, but did not show improvements so clonazepam was added with it.
Carbamazepine and trihexyphenidyl	Kwak et al., 2019 [12]	Male/13	Stage III	N/A as it served as supportive therapy	After three years, his symptoms were no longer noticeable.
Valproic acid	Campbell et al., 2005 [7]	Male/16	Stage III	750 mg / dose	No noticeable seizures during the treatment period. Later died due to acute respiratory distress syndrome (ARDS) and renal failure.
	Demirbilek et al., 2005 [21]	Male/10	Stage II	N/A	Myoclonic jerks persisted and became more abundant.
Valproic acid and clonazepam	Campbell et al., 2005 [7]	Female/6	Stage III	N/A	Improved the condition of myoclonic seizures.

Complementary Therapies

Complementary therapies may not be scientifically effective but can ease the psychological imbalance of the family members around the patient (Table 6). Since there is no protocol for treatment, many people go to extra lengths to incorporate therapies like hypnosis. Though it is not practical, it is a different form of therapy that impacts people around the patient than the patient themselves. When family members are at ease in their minds, supportive care of the patient would be better quality as their emotional distress will be milder. 

**Table 6:** Complementary therapies SSPE - subacute sclerosing panencephalitis

Other complementary therapies	Reference	Description of main methodology	Highlighted evidence
Alternative medicine (incense, incantations, and herbs)	Işıkay et al., 2017 [22]	Survey to fill out background of parents. Main objective was to understand the relationship between parents of SSPE patients and doctors, as well as gain an understanding of the perspective of different forms of treatment for fatal conditions.	13/29 parents of SSPE patients informed their doctor about using alternative medicine. Socio-economic class and level of education was a main reason why SSPE patients tend to look for more “spiritual“ treatments. The results vary based on culture.
Flupirtine	Tatlı et al., 2010 [23]	Flupirtine induces the opening of potassium channels in neurons and is an anti-apoptotic agent.	No conclusion. The paper hypothesizes that it will stop the spread of SSPE or slow down the progression.

Miscellaneous Treatments

These are treatments that did not have as many recent studies behind them; however, this does not make them unreliable. Rather, these are unpopular treatment plans due to the proposed risks or lack of effectiveness. Despite being disregarded by SSPE researchers thus far, they hold the potential to become incorporated into the future treatment plans for SSPE (Table 7).

**Table 7:** Miscellaneous treatments SSPE - subacute sclerosing panencephalitis, MSC - mesenchymal stem cells

Other pharmaceutical therapies	Reference	Sex/ age at onset	Patient SSPE stage at time of treatment	Dosage of treatment	Progress of treatment
Ketogenic diet	Bautista et al., 2003 [24]	Male/9	Stage I	750 calorie diet; calculated to sustain urine ketones at a level of -8.0 × 10^-9^ m^3^ kg	Within two weeks, myoclonic jerks stopped. After six weeks, the patient became more cognitively slow. Following three months of treatment, myoclonic jerks reappeared.
Immunoglobulin therapy (including valproic acid, levetiracetam, carbamazepine)	Har-Even et al., 2011 [15]	Male/16	Stage I	N/A	Determined to be ineffective. Deteriorated to stage IV and ultimately died.
Stem cell therapy	Kuşkonmaz et al., 2015 [25]	Male/9	Stage III	Eleven intravenous and eight intrathecal MSC infusions between two to eight month intervals over three years	Stable
		Male/11	Stage II	Two intravenous and intrathecal infusions at two month intervals	Progression then died
		Male/7	Stage II		Progression
		Male/9	Stage II	One intravenous and intrathecal MSC application	Progression and motor improvement

Discussion

The onslaught of the COVID-19 pandemic has forever changed research. Safety precautions to prevent the spread of the disease have negatively affected the progression of research projects, including suspensions of clinical trials and limited access to laboratory equipment. With these restrictions, shifting to research regarding COVID-19 had become attractive. This has also been a result of the combination of increased media exposure and the overwhelming monetary support to confront the root of the disease. 

Though the overwhelming number of papers had a positive impact on the pandemic, the quality of peer reviews of published papers has been compromised due to being unable to cope with the influx of papers. This has led to an increased number of misleading findings published in reputable journals. Pandemic concerns have also prevented the full potential of recruitment in journals and as subjects in research projects, aggravating the possibility of academic malpractice to forge findings [26].

Another problem has been the lack of papers that are published in regards to subjects that are not related to COVID-19. Cases of measles, the precursor to SSPE, have become more prevalent worldwide, despite it being preventable. The pandemic had limited immunization services, which prevented millions of people from getting their measles disease, increasing their susceptibility to being infected with measles. Despite an increase in potential test subjects in trials regarding SSPE, the safety precautions for COVID-19 limit any in-person activities, which is vital for discovering an effective treatment for SSPE [27].

Categorized into groups A to G, treatment methods were inputted into the results section based on how applicable the data is based on the quantitative observations provided. Group A was the interferon-related therapies, and only from the step of the organization of the data, it was found that interferon was often used in combined therapies. The treatments of interferon alpha, interferon alpha-2a, and interferon alpha-2b used individually provided a small margin of success rate, and they were rather ineffective despite showing initial progress. Successful treatment was the combined interferon alpha, inosine pranobex, and lamivudine in a randomized controlled trial by Aydin et al. [8]. The mortality rate was lower; three out of 19 compared to six out of 13 in the controlled group. Hosoya et al. reported ideal results with the combined treatment of interferon-alpha, ribavirin, and inosine pranobex with three patients who showed progress and two who remained stable. There were no adverse effects, and the patient’s quality of living was better. Interferon alpha and inosine pranobex showed very conflicting results as one patient lived for longer than thirteen years, and others deteriorated quickly or remained stable [9]. This treatment definitely had a big conflict of interest as the results were very contrary to one another. From the report by Kwak et al., it can be determined that the reason for the patient living for thirteen years is due primarily to the patient’s natural internal interventions, and the treatment of interferon alpha and inosine pranobex was more supportive [12]. 

Inosine pranobex did not show promising results. In one case, after discontinuing the treatments, the patients died due to respiratory illnesses. Cruzado et al. reported a female patient who received 100 mg/kg daily went into a vegetative state at 20 months and then died at 28 months [16]. In the report by Gokcil et al., after administering 50 to 100 mg/kg of body mass daily, the patient showed the same result by experiencing deteriorating conditions [28]. Nasirian et al. observed more positive results than others using this method. In four of 16 cases, the disease progression stopped, six cases exhibited slow progression and in six others the drug effect stopped completely [29]. Furthermore, out of the six patients who exhibited slow progression, three lived for an extra four years, two patients for up to seven years, and one surprisingly lived for an extra ten years. The definitive reason why the six patients who showed slow progression gained prolonged life is uncertain, but it could also be due to genetic progression. 

Another common pharmaceutical is ribavirin for treating SSPE. Tomoda et al. observed slow progression in seven out of ten patients, though the gender of the patients was not indicated [30]. Since males are more likely to be diagnosed with SSPE, it would have been important to know the sex to find a trend regarding which sex is most likely to react positively to treatments [31]. Amantadine was not found to be a promising treatment individually because Nasirian et al. found 10 of 14 patients reacting ineffectively to the treatment despite there being a cessation in one patient. 

Following the discussion of therapies for directly mitigating the progression of SSPE, symptomatic treatments must be discussed, beginning with anticonvulsants. Carbamazepine is a drug effective for stopping seizures, but in many of the reported cases described, it combined with trihexyphenidyl stops myoclonus [32]. Carbamazepine shows the most success when combined with other treatments. Valproic acid had contrary results as the stage III patient who had contracted SSPE at sixteen years old did not show any signs of seizures during the treatment, while a stage II patient at the age of ten had persisting myoclonic jerks [33, 34]. The second case by Demirbilek et al. did not provide dose information which could have been a contributing factor to the reason why one treatment was more effective. The age and stage of the disease may have also been environmental factors that made treatment more beneficial than the other [21]. 

Complementary therapies, in this report, are those that have theoretical backing to be implemented with combined therapy but are not commonly used. Alternative medicine is one that shows surprising results as the majority of people in lower socio-economic groups had opted for “spiritual”, including incense and incantations. The flupirtine is in the theoretical stage and has not been experimented with recently. This may be due to the demand for success and the pressure on physicians and scientists to test the most effective treatments rather than experiment with potentially beneficial treatments. However, fusion inhibitor peptides prevent trans-neuronal viral spread, which is the quantitative backing behind the use of this treatment method and the potential for it to bring a cessation to the viral spread of SSPE in the central nervous system [35].

The measles vaccination was not applicable to the results section but was included to discuss the main issue for the rising trend in SSPE cases due to non-vaccinators disrupting her immunity. The ketogenic diet had lacking documentation but proved to be somewhat useful [36]. Due to the nature of the diet, it may impact other therapeutic treatments, which is why it is not combined with other treatment plans, and there is little documentation on it. Unfortunately, for miscellaneous therapies, immunoglobulin therapy proved to be unsuccessful, and not many reports were recorded due to reports before 1999 showing similar results [37]. Surprisingly, the results for the miscellaneous treatments such as stem cell therapy showed potential as one case study showed that three out of four patients progressed, and out of the three, only one of them died due to respiratory complications [38]. Stem cell research is not an accepted form of treatment for SSPE, and despite having the potential to cure patients, it is a risky treatment plan that requires a large sample size to provide valid results, both of which cannot be achieved due to the already risky condition of SSPE patients as well as the impracticality expense-wise of collecting such a large group. 

Summary of evidence

The most evident trend in the data is that patients diagnosed with stage II of SSPE before treatment or lower tend to have a higher success rate. Another trend is that all treatments last for a certain period of time before the treatment becomes ineffective, even when the dose is increased. This window of effectiveness varies from person to person. Subacute sclerosing panencephalitis (SSPE) is a slowly progressing disease and commonly causes death after one to three years if measles infections are left untreated [39]. The drugs discussed in this research have implied that these types of therapies given alone or in combination halt the progression of the disease and can prolong life, but their long-term effects on individuals are unknown. In conjunction with previous reports, within the past five years, there has been an increase in SSPE due to decreasing rates of vaccination [40]. This preventable disease has the potential to be eradicated without the discovery of a cure if vaccination becomes compulsory in every region, but until then, those suffering from SSPE can be advised to follow the best treatment method discussed in this report.

Limitations

The findings of this study have to be seen in the light of some limitations. There was a randomized trial referenced, which is considered empirical findings as there was no substantial proof of treatment. As students, accessing papers with positive findings was difficult since most were locked, unavailable, or payment was required to access the full article. On the other hand, papers with adverse findings are usually easier to access because it demonstrates the severity of SSPE and emphasizes that prevention is important, discouraging the research of a cure. Some research required a level of a high degree of scientific literature understanding and results could have been misinterpreted due to a lack of time dedicated to understanding the articles. Overall, the main limitation would be the inability to validate the findings of the referenced reports. 

Further enhancements

To improve this research, the data collection should include the country each report originated from to determine which treatments could be more effective in certain countries over others. This would help gain an insight into the global SSPE cases and shed insight on the popular hypothesis of genetics being linked to how some people may be more susceptible than others to the disease, or it might provide a clinical profile as to how environmental factors (region of habitation, socio-economic status, etc.) may make a patient more prone to the disease than others. These findings can then aid in deciding where vaccination programs should be held.

## Conclusions

Before starting the research, it was a known fact that there is currently no cure for SSPE. However, this report explored the many treatment options that are in use, and many controversial ideas were analyzed. From the research, it can be discerned that certain treatments, such as the combination of interferon-related immunotherapy, need to be more heavily researched as they provide the most promise for future successful treatments. As more cases of SSPE arise, there will be more room for trials for hypothetical treatments with great potential like flupirtine, but intravenous interferon-alpha and ribavirin combined with oral Inosiplex is the most effective treatment despite controversy. Sequencing the DNA of many SSPE patients will also further improve the knowledge of whether treatments are more effective based on someone’s DNA, and it will aid in the prognosis of SSPE as future research may discover that genetics may make someone susceptible to SSPE. Currently, the most effective method to battle and eradicate SSPE from developed countries is widespread immunization as more and more people have the choice of vaccination when in actuality, it should be made compulsory. 
